# Absence of a Major Role for the Snai1 and Snai3 Genes in Regulating Skeletal Muscle Regeneration in Mice

**DOI:** 10.1371/currents.md.e495b27ee347fd3870a8316d4786fc17

**Published:** 2013-11-08

**Authors:** Christine R. Norton, Ying Chen, Xiang Hua Han, Cara K. Bradley, Luke T. Krebs, Jeong Kyo Yoon, Thomas Gridley

**Affiliations:** Center for Molecular Medicine, Maine Medical Center Research Institute, Scarborough, Maine, USA; Department of Biomedical Engineering, Tufts Univeristy, Medford, Massachusetts, USA; Center for Molecular Medicine, Maine Medical Center Research Institute, Scarborough, Maine, USA; Genea BioMedX, Sydney, New South Wales, Australia; Center for Molecular Medicine, Maine Medical Center Research Institute, Scarborough, Maine, USA; Center for Molecular Medicine, Maine Medical Center Research Institute, Scarborough, Maine, USA; Center for Molecular Medicine, Maine Medical Center Research Institute, Scarborough, Maine, USA

## Abstract

The Snail gene family encodes DNA-binding zinc finger proteins that function as transcriptional repressors. While the Snai1 and Snai2 genes are required for normal development in mice, Snai3 mutant mice exhibit no obvious abnormalities. The Snai3 gene is expressed at high levels in skeletal muscle. However, we demonstrate by histological analysis that Snai3 null mutant mice exhibit normal skeletal muscle. During hindlimb muscle regeneration after cardiotoxin-mediated injury, the Snai3 null mice exhibited efficient regeneration. To determine whether the Snai3 gene functions redundantly with the Snai1 gene during skeletal muscle regeneration, we performed hindlimb muscle regeneration in mice with skeletal muscle-specific deletion of the Snai1 gene on a Snai3 null genetic background. These mice also exhibited efficient regeneration, demonstrating that there is no major role for the Snai1 and Snai3 genes in regulating skeletal muscle regeneration in mice.

## Introduction

The Snail gene family encodes zinc finger proteins that function as transcriptional repressors 1 ^,^ 2. Three members of the Snail gene family have been described in mammals, encoded by the *Snai1* (also termed *Snail*), *Snai2* (*Slug*), and *Snai3* (*Smuc*) genes. While the *Snai1* and *Snai2* genes and proteins have been studied extensively in both mice and humans, much less is known about the functions of the *Snai3* gene. *Snai3* (originally termed *Smuc*, for *S*nail related gene from skeletal *MU*scle *C*ells) was isolated using a degenerate PCR-amplification protocol as a Snail family gene expressed in adult mouse skeletal muscle 3. Northern blot analysis revealed that the *Snai3* gene was highly expressed in adult mouse skeletal muscle and thymus, at lower levels in adult heart, lung and spleen, and was also expressed during embryogenesis 3. Analysis by in situ hybridization during mouse embryogenesis revealed that *Snai3* transcripts were first observed at embryonic day (E)13.5 in skeletal muscle and diaphragm 4. At E15.5, in addition to skeletal muscle and diaphragm expression, *Snai3* transcripts also were expressed in the thymus. Skeletal muscle and thymus remained the dominant sites of *Snai3* expression through the early postnatal period 4. However, both our laboratory 5 and the Weis laboratory 6 described recently the absence of an obvious phenotype in *Snai3* null mice. Here we report the analysis of *Snai3* mutant mice, and of *Snai1/Snai3* double mutant mice, using the cardiotoxin injury model of hindlimb skeletal muscle regeneration.

## Materials and methods

**Mice**

Generation and genotyping of the *Snai3^null^* 5, *Snai3-EYFP* 5 and *Snai1-flox* 7 mice have been described previously. *Myf5-Cre* mice 8 were obtained from the Jackson Laboratory, and *Mef2c-73k-Cre* mice 9 were obtained from Dr. Brian Black. All mouse protocols followed the guidelines of the US National Research Council Guide for the Care and Use of Laboratory Animals, and were approved by the Maine Medical Center Institutional Animal Care and Use Committee.

**Hindlimb regeneration assay, histological analyses, and skeletal preparations**

Tibialis anterior (TA) muscles of anesthetized mutant and control littermate mice at 11 to 18 weeks of age were injected with 100 ul of 10 uM cardiotoxin (Naja mossambica cardiotoxin; catalog number C9759, Sigma-Aldrich). Mice were euthanized 10 to 12 days after injection to collect the injured TA muscles for histological analysis. Some TA muscles were embedded in paraffin, sectioned at 7 um, and stained with hematoxylin and eosin. Other TA muscles were snap frozen and cryosectioned before staining with hematoxylin and eosin. All regeneration experiments were repeated at least three times. Myofiber cross sectional area was measured using Zeiss Axiovison software, and differences of means of the genotype groups were tested for statistical significance using the two tailed, unpaired Student's t test. Alcian blue-alizarin red-stained skeletal preparations were generated as described previously 10. Mice were euthanized, skinned, eviscerated, and fixed in 100% ethanol (EtOH). They were then stained in 0.015% Alcian blue, 0.005% Alizarin red in 5% acetic acid/70% EtOH. Clearing was performed in 2% potassium hydroxide, followed by 1% potassium hydroxide/20% glycerol, after which they were brought into 80% glycerol for photography and storage. For conditional deletion experiments, efficient gene deletion in skeletal muscle by the *Myf5-Cre* and *Mef2c-73k-Cre* drivers was confirmed by quantitative PCR on genomic DNA isolated from the gastrocnemius or TA muscle.

## Results

***Snai3* null mutant mice exhibit normal skeletal muscle regeneration after injury**

We recently described the generation and analysis of two different null alleles of the *Snai3* gene, *Snai3^null^* and *Snai3-EYFP* 5. Due to the fact that the *Snai3* gene was originally cloned from mouse skeletal muscle RNA, and is expressed at high levels in that tissue, we paid particular attention to possible pathological changes in skeletal muscle in the *Snai3* mutant mice. Histological analysis of multiple skeletal muscles of *Snai3^null^/Snai3^null^* or *Snai3-EYFP/Snai3-EYFP* homozygous mice through 13 months of age did not reveal any obvious skeletal muscle pathology, such as muscle hypotrophy, aberrant muscle fiber size, centrally located nuclei, or infiltration of fibrotic or adipose tissue (Figure 1).

**Snai3null/Snai3null mice have normal skeletal muscle.**
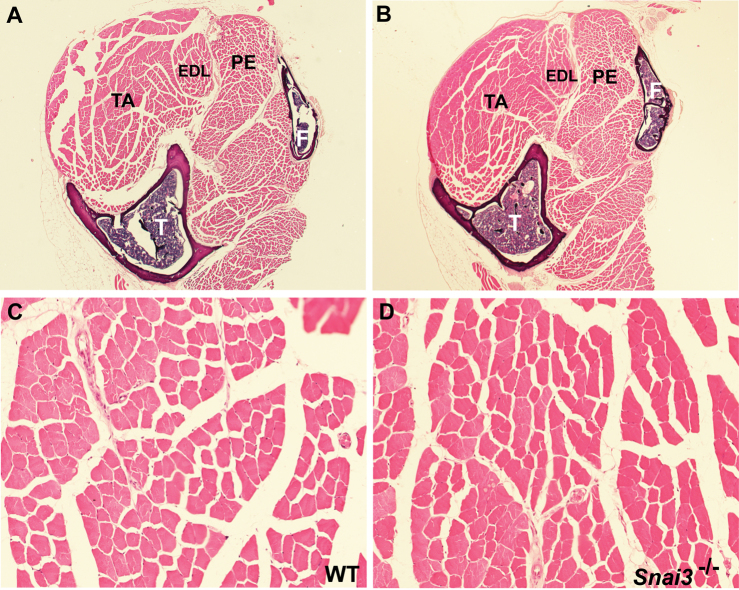
(A-D) Sections of the lower hindlimb of wildtype (A, C) and Snai3-null/Snai3-null (B, D) littermate mice at 13 months of age. No differences were observed between the Snai3 mutants and their wildtype littermates. Sections from paraffin-embedded legs were stained with hematoxylin and eosin. Abbreviations: EDL: extensor digitorum longus muscle; F: fibula; PE: peroneus longus muscle; T: tibia; TA: tibialis anterior muscle. Magnifications: (A, B) 2.5X; (C, D) 20X.

To additionally assess formation and integrity of the musculoskeletal system, we examined Alcian blue-alizarin red-stained skeletons of *Snai3^null^/Snai3^null^* or *Snai3-EYFP/Snai3-EYFP* homozygous and control littermate mice. No skeletal defects were observed in the *Snai3* mutant mice (Figure 2, and data not shown).

**Snai3null/Snai3null mice do not exhibit altered bone formation.**
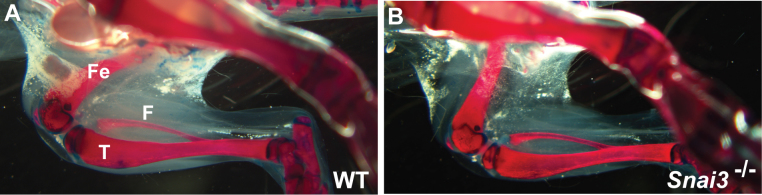
Skeletal preparations were stained with Alizarin red (to stain mineralized bone) and Alcian blue (to stain cartilage). No differences in skeletal formation were observed between the wildtype (A) and Snai3-null/Snai3-null (B) littermate mice. F: fibula; Fe: femur; T: tibia.

To assess whether conditions of stress might reveal a requirement for *Snai3* gene function in skeletal muscle, we tested the ability of the hindlimb Tibialis anterior (TA) muscle of *Snai3* mutant and control littermate mice to regenerate after injury, using the standard cardiotoxin injury model. Both TA muscles of *Snai3^null^/Snai3^null^* or *Snai3-EYFP/Snai3-EYFP* and control littermate mice were injected with cardiotoxin, and the TA muscles were isolated 10 to 12 days after injury for histological analysis. These studies did not reveal substantive differences in regeneration between *Snai3* ^null^/Snai3^null^ or *Snai3-EYFP/Snai3-EYFP* homozygotes and their heterozygous and wildtype control littermates. As assessed by the presence of centrally located nuclei in the myofibers, both *Snai3* mutant homozygotes and wildtype control littermates exhibited extensive myofiber regeneration after cardiotoxin injury (Figure 3A, B). The mean cross sectional area of regenerating myofibers did not differ significantly between *Snai3* mutant homozygotes and wildtype control littermates (Fig. 3C). Some, but not all, *Snai3* mutant homozygotes exhibited a small amount of fibrosis in the regenerating TA muscle. However, TA muscle regeneration still proceeded efficiently in the *Snai3* mutant mice.

**Snai3null/Snai3null mice exhibit no obvious defects in skeletal muscle regeneration.**
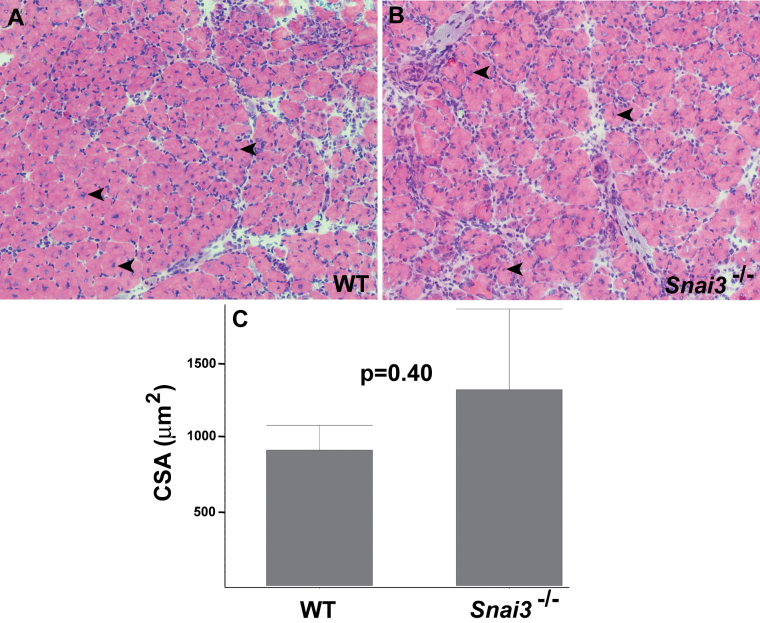
Hematoxylin and eosin-stained cryosections of TA muscle from wildtype littermate (A) and Snai3-null/Snai3-null (B) mice 10 days after cardiotoxin-mediated injury revealed no obvious differences between the two genotypes. Arrowheads indicate examples of centrally-located nuclei in regenerating myofibers. Magnification: 20X. (C) Mean myofiber cross sectional area (CSA) did not differ significantly between the wildtype littermate (n=2) and Snai3null/Snai3null (n=3) genotypes.

**Skeletal muscle regeneration in *Snai1/Snai3* double mutant mice**

We have shown that during chondrogenesis (cartilage development) in mice, the *Snai1* and *Snai2* genes function redundantly, and that both *Snai1* and *Snai2* gene function must be removed to detect a phenotype during cartilage formation 10 ^,^ 11 ^,^ 12. The *Snai1* gene is induced during skeletal muscle regeneration 13, and a recent paper demonstrated that a Snai1-HDAC1/2 repressive complex bound and excluded the myogenic transcription factor MyoD from its targets 14. We therefore decided to test TA muscle regeneration in *Snai1/Snai3* double mutant mice. Since *Snai1^null^/Snai1* ^*nul*l^ homozygotes die early in embryogenesis 15, we utilized our *Snai1-flox* allele 7 and either the *Myf5-Cr*e 8 or *Mef2c-73k-Cre* 9 driver lines to perform skeletal muscle-specific deletion of the *Snai1* gene.

We generated both *Myf5-Cre/+; Snai1-flox/Snai1-flox; Snai3^null^*/*Snai3^null^* and *Mef2c-73k-Cre/+; Snai1-flox/Snai1-flox; Snai3^null^/Snai3^null^* (or *Mef2c-73k-Cre/+; Snai1-flox/Snai1-flox; Snai3-EYFP/Snai3-EYFP*) mice (referred to as *Snai1/Snai3* double mutant mice). Histological analysis of uninjured TA muscles from the *Snai1/Snai3* double mutant mice did not reveal any obvious defects, compared to control littermate mice lacking the *Cre* allele (i.e., *Snai1-flox/Snai1-flox; Snai3-EYFP/Snai3-EYFP* mice) (Figure 4A, B).

TA muscles of *Snai1/Snai3* double mutant mice and control littermate mice were injected with cardiotoxin, and the TA muscles were harvested 12 days after injury for histological analysis. The regenerating TA muscles of *Snai1/Snai3* double mutant and control Cre-negative littermate mice appeared identical histologically (Figure 4C, D). Both control *Myf5-Cre*-negative;* Snai1-flox/Snai1-flox; Snai3^null^/Snai3^null^* TA muscle (Figure 4C) and *Snai1/Snai3* double mutant TA muscle (Figure 4D) exhibited extensive myofiber regeneration after cardiotoxin injury. The mean cross sectional area of regenerating myofibers did not differ significantly between the two genotypes (Figure 4E). We conclude that the *Snai3* gene does not play a major role in hindlimb skeletal muscle regeneration in mice, even in combination with the related *Snai1* gene.

**Mice with skeletal muscle-specific deletion of the Snai1 gene on a Snai3 homozygous mutant background exhibit no obvious defects in skeletal muscle development or regeneration.**
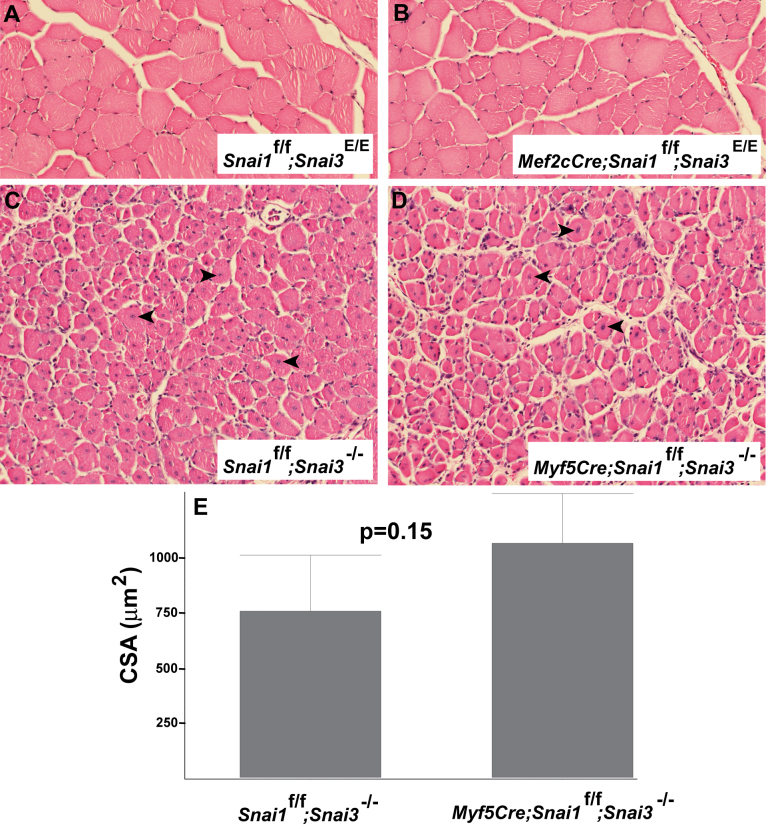
(A, B) Sections of uninjured TA muscle of control (Snai1-flox/Snai1-flox; Snai3-EYFP/Snai3-EYFP) (A) and Snai1/Snai3 double mutant (Mef2c-73k-Cre/+; Snai1-flox/Snai1-flox; Snai3-EYFP/Snai3-EYFP) (B) littermate mice at 5 months of age. (C, D) Sections of TA muscle 12 days after cardiotoxin-mediated injury from control (Snai1-flox/Snai1-flox; Snai3null/Snai3null) (C) and Snai1/Snai3 double mutant (Myf5-Cre/+; Snai1-flox/Snai1-flox; Snai3null/Snai3null) (D) mice. Arrowheads indicate centrally-located nuclei in regenerating myofibers. Sections from paraffin-embedded TA muscles were stained with hematoxylin and eosin. Magnification: 20X. (E) Mean myofiber cross sectional area (CSA) did not differ significantly between the control (n=3) and Snai1/Snai3 double mutant (n=4) genotypes.

## Discussion

Our results demonstrate effective skeletal muscle regeneration after cardiotoxin-mediated injury in *Snai3* homozygous mutant (*Snai3^null^/Snai3^null^* or *Snai3-EYFP/Snai3-EYFP*) mice. We further show that mice with skeletal muscle-specific deletion of the *Snai1* gene on a *Snai3* null genetic background exhibit the same general level of skeletal muscle regeneration as the *Snai3* mutant homozygotes. While our histopathological analyses cannot exclude minor regeneration defects in the *Snai3* single or *Snai1/Snai3* double mutants, it is clear that substantial muscle regeneration occurs after cardiotoxin-mediated injury in these mice.

A recent study utilized ChIP-Seq and gene expression analyses to demonstrate that a Snai1-HDAC1/2 repressive complex bound and excluded the myogenic transcription factor MyoD from its targets 14. These authors further showed that a regulatory network involving myogenic regulatory factors, Snai1/Snai2, and the microRNAs miR-30a and miR-206 acted as a molecular switch controlling entry into myogenic differentiation. It is possible that we did not observe a substantial effect on skeletal muscle development or regeneration in our experiments because our mice were wildtype at the *Snai2* locus.

In 2002, we participated in a study demonstrating that *Snai2* gene expression is induced during muscle regeneration, and that *Snai2-lacZ* homozygous null mice exhibit impaired hindlimb skeletal muscle regeneration 13. At the time of those studies, our *Snai2-lacZ* mice were on a mixed (129S1/SvImJ X C57Bl/6J) genetic background, and approximately 50% of homozygotes survived into adulthood 16. The remainder died postnatally from cleft palate. Since that time, we have maintained the *Snai2-lacZ* line as a heterozygous backcross to C57BL/6J mice, and our *Snai2-lacZ*/+ mice are now a congenic line on the C57BL/6J genetic background. We have found that on the C57BL/6J background virtually all *Snai2-lacZ/Snai2-lacZ* homozygotes now die in the early postnatal period, apparently as an increase in the penetrance of the cleft palate phenotype. We therefore were not able to test *Snai2-lacZ/Snai2-lacZ* mice, or compound mutants containing the *Snai2-lacZ* allele, in the current set of experiments. Further work, including the generation and utilization of a *Snai2-flox* allele for skeletal muscle-specific Snai2 gene deletion, will be required to remove the function of all three Snail family genes in skeletal muscle to definitively assess the requirement for Snail family genes during skeletal muscle development and regeneration.

## Correspondence

Thomas Gridley. Email: gridlt@mmc.org

## Competing Interests

The authors have declared that no competing interests exist.

## References

[1] A. Barrallo-Gimeno, M.A. Nieto, Evolutionary history of the Snail/Scratch superfamily, Trends Genet 25 (2009) 248-252. 10.1016/j.tig.2009.04.001 19427053

[2] C. Chiang, K. Ayyanathan, Snail/Gfi-1 (SNAG) family zinc finger proteins in transcription regulation, chromatin dynamics, cell signaling, development, and disease, Cytokine Growth Factor Rev 24 (2013) 123-131. 10.1016/j.cytogfr.2012.09.002 PMC3561489 23102646

[3] H. Kataoka, T. Murayama, M. Yokode, S. Mori, H. Sano, H. Ozaki, Y. Yokota, S. Nishikawa, T. Kita, A novel Snail-related transcription factor Smuc regulates basic helix-loop-helix transcription factor activities via specific E-box motifs, Nucleic Acids Res 28 (2000) 626-633. 10.1093/nar/28.2.626 PMC102498 10606664

[4] X. Zhuge, H. Kataoka, M. Tanaka, T. Murayama, T. Kawamoto, H. Sano, K. Togi, R. Yamauchi, Y. Ueda, Y. Xu, S. Nishikawa, T. Kita, M. Yokode, Expression of the novel Snai-related zinc-finger transcription factor gene Smuc during mouse development, Int J Mol Med 15 (2005) 945-948. 15870897

[5] C.K. Bradley, C.R. Norton, Y. Chen, X. Han, C.J. Booth, J.K. Yoon, L.T. Krebs, T. Gridley, The Snail family gene Snai3 is not essential for embryogenesis in mice, PloS One 8 (2013) e65344. 10.1371/journal.pone.0065344 PMC3675094 23762348

[6] P.D. Pioli, T.J. Dahlem, J.J. Weis, J.H. Weis, Deletion of Snai2 and Snai3 results in impaired physical development compounded by lymphocyte deficiency, PloS One 8 (2013) e69216. 10.1371/journal.pone.0069216 PMC3713067 23874916

[7] S.A. Murray, E.A. Carver, T. Gridley, Generation of a Snail1 (Snai1) conditional null allele, Genesis 44 (2006) 7-11. 10.1002/gene.20178 16397867

[8] M.D. Tallquist, K.E. Weismann, M. Hellstrom, P. Soriano, Early myotome specification regulates PDGFA expression and axial skeleton development, Development 127 (2000) 5059-5070. 10.1242/dev.127.23.5059 11060232

[9] A.B. Heidt, B.L. Black, Transgenic mice that express Cre recombinase under control of a skeletal muscle-specific promoter from Mef2c, Genesis 42 (2005) 28-32. 10.1002/gene.20123 15828002

[10] S.A. Murray, K.F. Oram, T. Gridley, Multiple functions of Snail family genes during palate development in mice, Development 134 (2007) 1789-1797. 10.1242/dev.02837 17376812

[11] Y. Chen, T. Gridley, The SNAI1 and SNAI2 proteins occupy their own and each other's promoter during chondrogenesis, Biochem Biophys Res Commun 435 (2013) 356-360. 10.1016/j.bbrc.2013.04.086 PMC3717576 23665016

[12] Y. Chen, T. Gridley, Compensatory regulation of the Snai1 and Snai2 genes during chondrogenesis, J Bone Miner Res 28 (2013) 1412-1421. 10.1002/jbmr.1871 PMC3663919 23322385

[13] P. Zhao, S. Iezzi, E. Carver, D. Dressman, T. Gridley, V. Sartorelli, E.P. Hoffman, Slug is a novel downstream target of MyoD. Temporal profiling in muscle regeneration, J Biol Chem 277 (2002) 30091-30101. 10.1074/jbc.M202668200 12023284

[14] V.D. Soleimani, H. Yin, A. Jahani-Asl, H. Ming, C.E. Kockx, W.F. van Ijcken, F. Grosveld, M.A. Rudnicki, Snail regulates MyoD binding-site occupancy to direct enhancer switching and differentiation-specific transcription in myogenesis, Mol Cell 47 (2012) 457-468. 10.1016/j.molcel.2012.05.046 PMC4580277 22771117

[15] E.A. Carver, R. Jiang, Y. Lan, K.F. Oram, T. Gridley, The mouse Snail gene encodes a key regulator of the epithelial-mesenchymal transition, Mol Cell Biol 21 (2001) 8184-8188. 10.1128/MCB.21.23.8184-8188.2001 PMC99982 11689706

[16] R. Jiang, Y. Lan, C.R. Norton, J.P. Sundberg, T. Gridley, The Slug gene is not essential for mesoderm or neural crest development in mice, Dev Biol 198 (1998) 277-285. 9659933

